# Participation in Complex and Social Everyday Activities Six Years after Stroke: Predictors for Return to Pre-Stroke Level

**DOI:** 10.1371/journal.pone.0144344

**Published:** 2015-12-10

**Authors:** Avvai Singam, Charlotte Ytterberg, Kerstin Tham, Lena von Koch

**Affiliations:** 1 Nyköpings Hospital, Nyköping, Sweden; 2 Karolinska Institutet, Stockholm, Sweden; 3 Karolinska University Hospital, Stockholm, Sweden; University of Brescia, ITALY

## Abstract

**Background:**

Long-term disability following stroke can lead to participation restrictions in complex and social everyday activities, yet information is lacking on to what extent stroke survivors return to their pre-stroke levels of participation.

**Objectives:**

The objectives of this study were to investigate the level of participation in complex and social everyday activities 6 years after stroke, to compare this with pre-stroke participation and to identify predictors of returning to pre-stroke levels of participation.

**Method:**

All patients admitted to Karolinska University Hospital's stroke units during a 1-year period were eligible to participate and 349 patients were recruited. Assessments were made at base-line, 3 months and 6 years using self-reported outcome measures. Participation was assessed using the Frenchay Activities Index (FAI). The 6-year score for each participant was compared to the pre-stroke score, both for the total score and for each domain (domestic chores, leisure/work and outdoor activities). Predictors of having the same or better level of participation at 6 years were identified using logistic regression.

**Results:**

At 6 years, 121 participants were followed up, 166 were deceased, 44 declined to take part and 18 could not be traced. At 6 years 84% could be described as active (FAI≥15). The same level of participation or better than pre-stroke was found in 35% of participants, in 65% the level was lower. Similar predictors were identified for achieving the same or better level of participation at 6 years for FAI total and the three domains; ability to walk without aids and a lower age at stroke onset, and perceived mobility, participation and recovery at 3 months.

**Conclusion:**

Six years after stroke, 35% of participants had the same or better level of participation as pre-stroke. Rehabilitation after stroke to improve walking ability and participation might improve long-term participation in complex and social everyday activities.

## Introduction

Stroke is one of the leading causes of long-term disability worldwide. In Sweden the incidence of stroke is about 25,000 per year [1], resulting in large demands on health and social services [1]. Most strokes (75%) occur in individuals over the age of 65. Despite a decreasing incidence rate, an ageing population [1] combined with better survival rates [1] is expected to lead to an increased prevalence and need for efficient rehabilitation and health care policies for people with stroke.

The physical, cognitive and psychological impairments resulting from stroke can lead to a large range of activity limitations and participation restrictions. The International Classification of Functioning, Disability and Health (ICF), based on a biopsychosocial model of disability, defines activity as “the execution of a task or action by an individual”; activity limitations are therefore considered difficulties an individual has in carrying out activities [2]. Participation is defined as “involvement in a life situation”; participation restrictions are problems experienced in this involvement [2].

A return to previous activities and way of living is of great importance for well-being, and often seen as a goal for rehabilitation [3–6]. A suitable outcome for investigation, therefore, is the level of participation in complex and social everyday activities e.g. domestic chores, leisure, work and outdoor activities. These are activities requiring more organisation, decision-making and interactions than everyday personal care activities.

To fully understand the consequences of stroke, the individual’s perspective on his/her functioning after stroke should also be assessed using valid and reliable patient-reported outcome measures. Furthermore, the importance of considering the individual’s perspective is recognised in the Swedish health care system where the Health Care Act requires that decision making and planning of medical care be carried out together with the patient [7].

Although stroke is acknowledged as a long-term condition most studies concerning participation after stroke have been short-term or cross-sectional and little is known about long-term outcomes. Some long-term stroke studies have, however, been carried out and have shown that five to ten years after stroke, at least 30% of survivors experience a reduced level of participation in complex and social everyday activities [8,9].

Similarly, few studies have been found in which predictors for participation in complex and social everyday activities five years post-stroke have been studied [9–12]. Higher age at stroke onset, more severe stroke, dependency in activities of daily life (ADL) (3 months post-stroke and at 5 years), suffering a recurrent stroke, depression and low cognitive function have been identified as predictors of decreased participation [9–12]. Studies with follow-up periods of two to four years have found the same predictors as above as well as an increased number of co-morbidities and decreased upper and lower extremity function (at hospital discharge) [13–14].

Only one study has been found in which participation in complex and social everyday activities pre-stroke was compared with long-term participation. The results showed significant participation restrictions post-stroke [15]. However, that study was cross-sectional and included 30 participants with large variations in the time since they had had their strokes. Their pre-stroke participation was only assessed retrospectively, which may affect the accuracy. More research using valid and reliable outcome measures with larger samples is therefore required to evaluate long-term participation and the extent to which people with stroke return to their pre-stroke levels of participation. It is essential to identify factors in the early post-stroke period (from stroke onset to six months) that predict long-term participation, in particular those that are modifiable, and to identify which individuals might be at a greater risk of decreased participation. This knowledge might be of use in the planning of the rehabilitation for the person with stroke and in the development of health or social services that aim to reduce restrictions in participation in complex and social everyday activities. It might also enable more correct prognostic information to be given to stroke patients and their families at stroke onset.

The aims of the study were:

1. To investigate the level of participation in complex and social everyday activities in individuals 6 years after stroke.
2. To investigate the extent to which individuals return to their previous level of participation in complex and social everyday activities 6 years after stroke.
3. To identify predictors of returning to previous levels of participation in complex and social everyday activities 6 years after stroke.

## Method

### Ethical permission

Ethical permission was applied for and then granted by the Regional Ethics Committee in Stockholm both for the original study and the 6-year follow-up study (applications: 2005/1462-31/3, 2011/1573-32 and 2012/428-32).

### Study design

A prospective, longitudinal study “Life after Stroke” with the aim of exploring rehabilitation needs of people with stroke was started in 2006.

### Study Population

Participants were recruited for the “Life after Stroke” study from Karolinska University Hospital in Stockholm, Sweden. The hospital had a source population of 26,000 (in 2006) and three stroke units. Inclusion criteria were a verified stroke diagnosis according to ICD-10 classification and admission to a stroke unit between 16^th^ May, 2006 and 15^th^ May, 2007. Detailed information on the project was given, with written informed consent obtained both at study inclusion (covering all assessments during the first year) and at 6 years.

### Data Collection and Measures

Clinically experienced occupational therapists and physiotherapists who had received training in the use of the measures carried out the data collection. Patients were first assessed while in the stroke unit within five days after stroke. Follow-up assessments were carried out at 3, 6 and 12 months, and then at 6 years post-stroke, usually at the participant’s home. Where necessary, a close relative or interpreter was present during data collection. In the present study, data from baseline, 3 months and 6 years were used.

Participation was measured with the Frenchay Activities Index (FAI), which contains 15 items representing three domains: domestic chores, leisure/work and outdoor activities [16]. Some initiative and planning is required to score on the items. A score of between 0 and 3 is given for each item based on how often the activity has been performed during the previous 3 or 6 months (depending on the item). Generally an individual with a total FAI score below 15 is considered as being inactive [8,11,12,14]. In some studies the active individuals are further dichotomised into two groups [11,12,14]. FAI was developed to give information on the level of activities seen pre- and post-stroke [16] and has good psychometric properties such as reliability [17] and validity [16,18] when used with people with stroke.

In the present study the total 6-year score for each participant was compared to their pre-stroke score to investigate whether they had been able to return to their pre-stroke level of participation. Participants who at 6 years had the same FAI score as pre-stroke or better were defined as having a favourable outcome, whereas those with a lower score were considered as having an unfavourable outcome. Analyses were made for the total FAI score and for each of the three domains (domestic chores, leisure/work and outdoor activities).

Data collected by interview included socio-demographic characteristics (age, sex) and socioeconomic status (self-defined level of private financing: insufficient, just sufficient, more than sufficient, level of education: secondary/sixth form or university). Information regarding the participants’ country was obtained from the Population Registration Authority. Information on stroke subtype and co-morbidity was obtained from medical records.

Stroke severity at stroke onset was classified using the Barthel Index (BI) as this has shown good agreement with other stroke severity measures [19]. The index includes ten personal care and mobility activities, each scoring 0, 5 or 10 points resulting in a score of 0–100, where a higher score reflects a greater degree of independence [20]. A score ≤14 was classified as severe, 15–49 moderate, and ≥50 as mild stroke.

The self-perceived impact of stroke at 3 months was measured using the Stroke Impact Scale (SIS). It assesses eight areas: strength, hand function, ADL, mobility, communication, emotion, memory and cognitive ability, and participation. A total of 59 items are scored from 1 to 5, an algorithm is then used to create total scores of 0–100 for each area, where 0 represents maximum impact, and 100 no impact. SIS also includes a separate question asking the individual to rate his/her recovery on a scale from 0–100 where 0 is not recovered at all and 100 is fully recovered. SIS has good reliability, validity and sensitivity [21].

Walking ability at stroke onset was assessed using the 10meter walk test [22]. Results were categorised according to the gait item in the Scandinavian Stroke Scale: walking without aids, walking independently with aids, walking with assistance and aids, or unable to walk [23].

### Statistical Analysis

To analyse differences between participants with favourable and unfavourable participation outcomes at 6 years, the Mann-Whitney U-test was used for numerical data and the Chi-squared test for categorical variables. The level of significance was set at p≤0.05.

Logistic regression was then used to identify predictors of participation at 6 years by creating a model of the relationships between independent variables at stroke onset or 3 months and a favourable participation outcome at 6 years in total FAI score and for each of the three domains respectively. The variables were tested for possible inclusion in the models if they were either a) statistically significant in the univariate analysis, b) of importance in earlier studies, c) modifiable by rehabilitation or d) reflected the participants’ perception of the impact of stroke. The models were created in a multivariate logistic regression with stepwise forward selection where variables with p≤0.05 were entered and those with p≥0.10 were removed. When modelling the number of variables included at the same time was restricted to those allowed for by the sample size. The Hosmer—Lemeshow goodness-of-fit test was used on the resulting model to calculate the predictive accuracy of the model. A null hypothesis of there being no difference between observed and model-predicted values means that a non-significant result shows a well-fitting model [24]. The receiver operating characteristic curve was also used to determine the sensitivity and specificity of the models.

The SAS® System 9.3, SAS Institute Inc., Cary, NC, USA was used for the statistical analysis.

## Results

### The study population

The original study recruited 349 participants from 2006 to 2007, of which 121 remained at the 6-year follow-up. Of those not participating, 166 were deceased, 44 declined to take part and 18 could not be traced. The mean age of the participants in the present study at stroke onset was 63.4 years with a range from 24 to 85 years and at the 6-year follow-up the mean age was 69.4 years with a range from 30 to 91 years. Two thirds of the participants were men. The mean age of all 349 participants in the original study group at stroke onset was 72.4 years. The mean age of participants from the original study group who were deceased at six years was at stroke onset 78.2 years (SD 9.6). Further information on the study participants can be seen in Table 1.

**Table 1:** Background information on participants included in the original study (n = 349) and in the 6-year follow-up (n = 121).

Variable	Number (%) n = 349	Number (%) n = 121
*Medical history at inclusion*:		
Previous stroke	110 (32)	28 (23)
Transient ischaemic attack	12 (3)	5 (4)
Hypertension	145 (42)	62 (51)
Diabetes	58 (17)	18 (15)
Ischaemic heart disease	33 (9)	11 (9)
Atrial fibrillation	81 (23)	17 (14)
Employed/retired at six years	76 (22)/273 (78)	22 (20)/99 (80)
Civil status: living alone/together at six years	170 (49)/178 (51)	58 (48)/63 (52)

In the present study, the mean FAI score for all participants pre-stroke was 30.4 and at 6 years 27.1. The number of participants with a total FAI score <15 pre-stroke was 5 (4%) and at 6 years the number of participants had increased to 20 (16.5%). At 6 years post-stroke 42 (35%) participants had a favourable participation outcome whereas 79 (65%) had an unfavourable outcome.

### Factors associated with a favourable FAI outcome

Results from the univariate analyses of favourable and unfavourable outcome in FAI are depicted in Table 2. Participants with a favourable outcome were significantly younger at stroke onset (58.8 years) than those with an unfavourable outcome (65.8 years), p = 0.015. Self-perceived participation at 3 months according to SIS was significantly higher for participants with a favourable outcome (mean 75.4) than those with an unfavourable outcome (mean 64.8), p = 0.05; the same trend was seen in self-perceived recovery at 3 months (71.3 compared to 59.6), p = 0.01.

**Table 2:** Characteristics of the sample at six years after stroke and associations with favourable and unfavourable outcome.

Independent variables	All	Favourable participation outcome	Unfavourable participation outcome	p value
	n = 121	n = 42	n = 79	
Mean age^*a*^ years (SD)	69.4 (13.8)	58.8 (15.7)	65.8 (12.1)	0.01
Men/women (n)	79/42	28/14	51/28	0.12
Swedish/foreign-born (n)	97/24	36/6	61/18	0.27
*Private financing* ^*a*^				
Insufficient (ref) (n)	10	3	7	
Sufficient (n)	31	6	25	0.48
More than sufficient (n)	66	30	36	0.36
*Educational level*				
Secondary/sixth-form (n)	72	23	49	
Higher education (n)	40	16	24	0.39
*Stroke severity*				
Mild (n)	96	39	57	
Moderate/severe (n)	25	3	22	0.01
*Walking ability* ^*a*^				
Unable to walk or walks with aids and assistance (n) ref.	29	2	27	
Walks with aids (n)	16	3	13	0.24
Walks without aids (n)	61	34	27	<0.001
*Stroke Impact Scale* ^*b*^ mean (SD)				
Strength	73.6 (22.6)	80.9 (19.1)	69.8 (23.4)	0.02
Memory	83.0 (18.1)	87.0 (13.2)	81.0 (20.0)	0.11
Emotion	75.4 (17.5)	76.1 (15.2)	75.0 (18.3)	0.76
Communication	86.1 (20.4)	91.9 (12.7)	83.1 (23.0)	0.04
Activities of Daily Living	82.0 (21.1)	92.1 (12.0)	76.7 (22.9)	0.001
Mobility	83.0 (21.8)	92.9 (9.9)	77.8 (24.4)	0.002
Hand function	74.0 (30.8)	86.8 (18.3)	67.6 (33.8)	0.005
Participation	68.5 (25.8)	75.4 (24.5)	64.8 (26.0)	0.05
Recovery	63.6 (21.8)	71.3 (21.1)	59.6 (21.2)	0.01

^a^ = at stroke onset

^b^ = at 3 months

Results of favourable and unfavourable outcome for each of the three FAI domains are depicted in Table 3.

**Table 3:** Number (%) of participants with favourable /unfavourable outcome in each Frenchay Activities Index (FAI) domain at six years after stroke.

FAI domain	Favourable participation outcome n (%)	Unfavourable participation outcome n (%)
Domestic chores	62 (51.2)	59 (48.8)
Leisure/work	58 (47.9)	63 (52.1)
Outdoor activities	55 (45.5)	66 (54.5)

Results from the logistic regression for a favourable FAI outcome are shown in Table 4. When the total FAI scores were analysed being able to walk without aids and age at stroke onset were found to be predictors of a favourable outcome in participation 6 years after stroke. When the FAI domains were analysed the following predictors for a favourable outcome were identified, for the domestic domain a higher SIS mobility score, for the leisure/work domain lower age and a higher SIS participation score and for the outdoor activities domain lower age and a higher SIS recovery scale score. In Table 5 the area under the receiver operating characteristic curve, sensitivity, specificity and Hosmer-Lemeshow goodness of fit test results for each model are presented.

**Table 4:** Binary logistic regression for predictors of a favourable Frenchay Activities Index (FAI) outcome at six years after stroke.

FAI total/domain	Independent variables	Variable categorization	Odds Ratio Estimate (95%CI)		
Total	Walking ability^a^	Without aids vs unable to walk and with aids and support	15.52 (2.90–3.05)		
		With aids vs unable to walk and with aids and support	2.06 (0.23–18.42)		
Total	Age		0.94 (0.90–0.98)		
Domestic chores	SIS mobility^b^		1.06 (1.02–1.10)		
Leisure/work	Age^a^		0.93 (0.89–0.97)		
	SIS participation^b^		1.03 (1.00–1.05)		
Outdoor activities	Age^a^		0.94 (0.90–0.98)		
	SIS recovery scale^b^		1.03 (1.01–1.06)		

^a^ = at stroke onset

^b^ = at three months

**Table 5:** Area under the receiver operating characteristic curve, sensitivity, specificity and Hosmer-Lemeshow goodness of fit for Frenchay Activities Index (FAI) total and each domain.

FAI total/domain	Area under ROC^a^ curve	Sensitivity	Specificity	Goodness of fit test^b^
Total	0.821	70.0%	78.4%	0.402
Domestic chores	0.719	52.7%	54.7%	0.436
Leisure/work	0.755	64.7%	68.4%	0.386
Outdoor activities	0.744	64.6%	65.0%	0.977

^a^ = receiver operating characteristic curve

^b^ = Hosmer-Lemeshow goodness of fit test

## Discussion

### Participation in complex and social everyday activities

This study presented information on stroke survivors and the extent to which they returned to their previous level of participation in complex and social everyday activities 6 years after stroke in a new way. Earlier studies have only compared group averages. It also identified that both being able to walk without aids and age at stroke onset were of importance for participation after 6 years. For participation in domestic chore activities a higher SIS mobility score at three months was identified as a predictor for a favourable outcome, for leisure/work activities lower age and a higher SIS participation score and for outdoor activities lower age and a higher SIS recovery scale score.

The mean FAI score 6 years after stroke was found in the present study to be 27.1, which is similar to the mean score found in previous research [10]. Another study reported a lower mean score after four years possibly because there was a larger proportion of participants with moderate or severe strokes (35%) compared to the present study (21%) [14]. The mean score in the present study lay just below the ranges reported for a general population in England [25], the difference being surprisingly small. Possible explanations for this might be the large number of participants in the present study with a mild stroke, as well as that the older and those with more severe strokes were probably deceased. The activities included in FAI might be of the sort that are relatively easy to return to; however, differences might exist in how the activity is performed between people with stroke and the general population. The proportion of younger participants (<65 years) in the present study was 27% and younger individuals might have both better potential for recovery and be able to achieve high FAI scores even though not fully recovered from their strokes.

Previous research using FAI as an outcome measure has often used a cut-off score of <15 to define participants as inactive, with research finding that after four years 29% of the participants fell under this definition [14], and after five years 21% [11] to 40% [8] did. In the present study only 16.5% were found to be inactive, which could possibly be explained by the low percent (4%) of inactive participants pre-stroke (pre-stroke levels are not given for the previous studies [8,11,14]), differences in the health and social care available in the study contexts [26,27], cultural backgrounds [8,28] or socioeconomic factors [9,28].

In the present study a new way of analysing participation was applied, and it was shown that 35% of the participants were still as active or more so in complex and social everyday activities as before stroke occurrence. The analysis of each FAI domain showed that 51.2% of the participants were still as active or more so in domestic chores, 47.9% in leisure/work activities and 45.5% in outdoor activities. It is conceivable that participation is lower in outdoor activities as these activities are generally likely to be more demanding than domestic chores. Previous research comparing participation pre- and post-stroke found that 83% of participants retained participation in activities 2 to 14 years after stroke [15]. The higher percentage of favourable outcomes in participation in that study could possibly be explained by the more demanding inclusion criteria e.g. the participants were required to be medically stable, living in the community, understand two to three stage commands and sustain adequate attention for testing, that rendered a sample that is likely to represent a smaller proportion of the stroke population than the sample in the present study. In contrast, 65% of the participants in the current study did not return to their previous levels of participation. Of these, 75% (59 individuals) would have been classified as active using a cut-off score of 15, though they in fact had lower levels of participation in complex and social everyday activities than pre-stroke. This might be because of physical, cognitive and psychological impairments due to stroke [9–15], other co-morbidities [13], age [8–11,13,14], or gender [8,9].

### Predictors of participation

Predictors of a favourable outcome were found to be walking ability, SIS mobility (including several aspects of walking such as walking with balance, walking fast and climbing stairs), SIS recovery, SIS participation and age.

Walking ability and mobility might be modifiable factors and the importance of having a good walking ability is useful information for the planning of rehabilitation. Rehabilitation should be directed with regard to this aspect of functioning with interventions to improve walking without aids and mobility while not increasing the risk of falling. Being able to walk independently but with an aid did not result in a significantly higher level of participation, possibly because of environmental barriers. Although walking ability is mostly determined in the early post stroke period there are studies indicating that long-term rehabilitation might result in small improvements, which might be of importance for the individual patient [29,30]. For individuals unable to walk without aids despite rehabilitation, training in the appropriate environment might enable increased participation in complex and social everyday activities. Active patient participation has, for example, been found to be promoted in the home environment [31].

Predictors of a favourable outcome in the leisure/work and outdoor activities domains were SIS participation and self-perceived recovery indicating the importance of being able to participate in meaningful complex and social activities in the early post-stroke period.

Age was also found to be an important predictor in a five-year study where a one-year increase in age was associated with a 0.28 point decrease in FAI-score [10], as well as in studies with follow-up periods of two to four years [13,14]. The importance of age for participation in complex and social activities after stroke is especially relevant in an ageing population and needs to be considered when planning future health and social services. Furthermore, resources need to be made available to facilitate participation for the oldest stroke survivors.

Cognitive impairment was not a significant predictor of participation in complex and social everyday activities in the present study, although this was the case in earlier studies [10–12]. This may be due to the use of self-reported SIS memory and thinking as a proxy for a performance-based measure in the model. Hence more studies are warranted on the predictive value of cognitive function, when objectively measured, on long term favourable outcome in participation in complex and social everyday activities after stroke. Furthermore, other variables of importance for the prediction of favourable outcome need to be identified since the final model did not predict all participants with a favourable outcome. Additionally the participants' satisfaction with how the activities are performed and their perception of the quality need to be considered in future studies since these aspects were not addressed in the present study.

### Background factors of the study population

The mean age of the participants in this study (69.4 years at follow-up, 63.4 years at stroke onset) and the prevalence of co-morbidities were similar to those in other studies [9–14,32]. Most participants suffered a mild stroke (79.3%) as classified by the BI; this is similar to other studies (about 80% [8], 64.6% [10]) suggesting that the participants in the present study were a representative sample for people with stroke 6 years after occurrence.

The mean age of the sample in the current study at stroke onset was lower than that in the original study, as the older participants in the original work were now deceased. The mean age for all stroke patients in Sweden in 2006 was 76 years [33], which is slightly higher than in the original study group. A possible explanation might be because of socio-demographic differences in the study population, which came from an urban area where the mean age is lower than in rural areas [34].

There were several variables e.g., civil status that in the present study were not predictors of a favourable outcome. Further studies are warranted to explore the influence of contextual factors on long-term participation in complex and social everyday activities after stroke.

### Methodological discussion

Participation in complex and social everyday activities has been assessed using the total FAI score. It is possible that participants, although being described as active, have not been able to return to previous activities but participate in other activities. Future research could investigate changes in participation in the 15 different items included in the scale. It is also possible that participants are active in ways not covered by FAI, such as volunteer work, making telephone calls, watching TV, listening to the radio and using a computer [35].

The definition of a favourable outcome can be questioned, as it might be expected that participation declines solely because of age. However, FAI results for different age groups in the general population showed maximum scores in the age-groups 45–54 and 55–64, and then only small decreases until an age of >85 years [25]. In the present study, 27% of the participants were 64 years or younger, and 12% were 85 years or older.

### Strengths and limitations of the study

The strengths of the study include a) the method of participant inclusion whereby all stroke patients admitted to Karolinska University Hospital’s stroke units were eligible to participate, b) the use of face-to-face interviews for data collection c) the use of valid and reliable outcome measures covering several areas of functioning, d) the longitudinal design allowing identification of predictive factors, e) the inclusion of patient-reported outcome measures, f) that patients with co-morbidities were not excluded and g) the analysis of each FAI domain separately.

A limitation of the study is the number of participants who declined to take part in the 6-year follow-up, which might affect the external validity of the final model.

The study aimed to include all stroke patients admitted to a stroke unit but it is possible that patients with a milder stroke were missed because of their very short stays in the stroke unit. Even patients with very severe strokes might only have had short stays [36] or may not have been able to participate.

## Conclusion

Six years after a stroke 84% could be described as being active in complex and social everyday activities although only 35% were as active as they were before stroke. The results also showed that being able to walk without aids is of importance for participation and should be considered when planning rehabilitation. Further studies are needed to identify additional predictors for maintained long-term participation as well as the participants' satisfaction with how the activities are performed and their perception of the quality.

## Supporting Information

S1 Dataset Independent variables, columns A-N: Foreign-born (1 = yes, 2 = no), Walking ability (1 = Unable to walk or walks with aids and assistance, 2 = Walks with aids, 3 = Walks without aids), Private financing (1 = Insufficient or sufficient, 2 = More than sufficient), Educational level (1 = Secondary/sixth-form, 2 = Higher education), SIS, column E-M (mean), Stroke severity (1 = mild, 2 = moderate/severe). Dependent variables, columns O-R (1 = favourable outcome, 0 = Unfavourable outcome).
(XLS)
